# Male spiny frogs enter the underwater battlefield with loose skin exhibiting enhanced penetration of capillaries into the epidermis

**DOI:** 10.1186/s40851-023-00219-4

**Published:** 2023-10-06

**Authors:** Shuang Xu, Qiang Dai, Yuchi Zheng

**Affiliations:** 1 Chengdu Institute of Biology, Chinese Academy of Sciences, #9 of Section 4, Ren-Min-Nan Road, Wuhou District, Chengdu, 610041 Sichuan Province China; 2 University of Chinese Academy of Sciences, Beijing, 100049 China; 3 Key Laboratory of Southwest China Wildlife Resources Conservation (Ministry of Education), China West Normal University, Nanchong, 637009 Sichuan China

**Keywords:** Exchange surface, Hemoglobin density, Oxygen partial pressure, Erythrocyte size, Male‒male fighting, Pointed weapon, Loose skin, Combat trait, Capillary density, Underwater vocalization

## Abstract

The skin has multiple functions, and capillaries can penetrate the epidermis to shorten the diffusion path while allowing maintenance of overall epidermal thickness for nonrespiratory roles. However, a method for quantifying the capillary penetration extent is lacking. Such a method may facilitate making comparisons and detecting associations, potentially making the extent a useful variable in biological studies. We quantified the extent as the ratio of the average minimum thickness of epidermis overlying each capillary to the average epidermal thickness along a skin section and then explored its performance in the Emei mustache toad, *Leptobrachium boringii*, a species in which breeding males with loose skin call and fight each other with maxillary spines underwater. The ratio showed informative associations with other variables, such as perfused capillary density. It displayed small intragroup variation and could be more sensitive than other variables in revealing structural differences in the skin. The ratio estimates were lowest and were correlated with epidermal and *stratum compactum* thicknesses in breeding males, i.e., a covariation but not reinforcement against stabbing, constituting early evidence consistent with the increased extensibility of loose skin conferring a defensive advantage during combat in amphibians. In addition, our results lead to the hypothesis that high hemoglobin density along subepidermal capillaries favors the maintenance of low blood partial oxygen pressure and hence increases cutaneous oxygen uptake. We also provide evidence supporting the new idea that the cooccurrence of loose skin and underwater calling found in some frogs can be explained by the latter benefiting from a large functional respiratory surface area. Awareness of the usefulness of the ratio may promote its application and the quantification of the penetration. Regarding exchange surface design, these findings for *L. boringii* imply a case in which looseness increases surface area as well as prevents damage.

**Supplementary Information:**

The online version contains supplementary material available at 10.1186/s40851-023-00219-4.

## Background

Respiration is not restricted to specialized organs such as fish gills and spider book lungs but is also possible when the respiratory medium is separated from the blood or cells by a relatively thin membrane. Cutaneous gas exchange is widespread in vertebrates and occurs in other animal groups [1, 2]. As reported in some amphibians and fishes, skin capillaries may penetrate the epidermis, shortening the diffusion path length to, for instance, less than 10 μm [3–9]. This arrangement facilitates cutaneous respiration while maintaining the overall thickness of the epidermis for nonrespiratory roles such as protection [1, 10–12]. However, a method for measuring the extent of capillary penetration into the epidermis is lacking in the literature. Such a method may contribute to comparing and linking results and to uncovering associations between penetration and other factors or variables.

The capillary penetration extent can be quantified by the ratio of the average minimum overlying skin thickness across capillaries to the average epidermal thickness along a skin section. In a micrograph of the section, a path for approximating the minimum thickness of the skin overlying a capillary may be readily determined manually. To estimate the average thickness of a certain length of epidermis from the micrograph, one can divide the area of the epidermis by the length of a freehand line [13, 14] drawn along the epidermis. In the past two decades, with charge-coupled devices and image processing software (e.g., ImageJ [15]), it has become convenient to perform such measurements. An exploration of the relationships between the capillary penetration extent and other factors may inspire efforts to expand our understanding of the coordination of different functions of vertebrate skin.

**Fig. 1:**
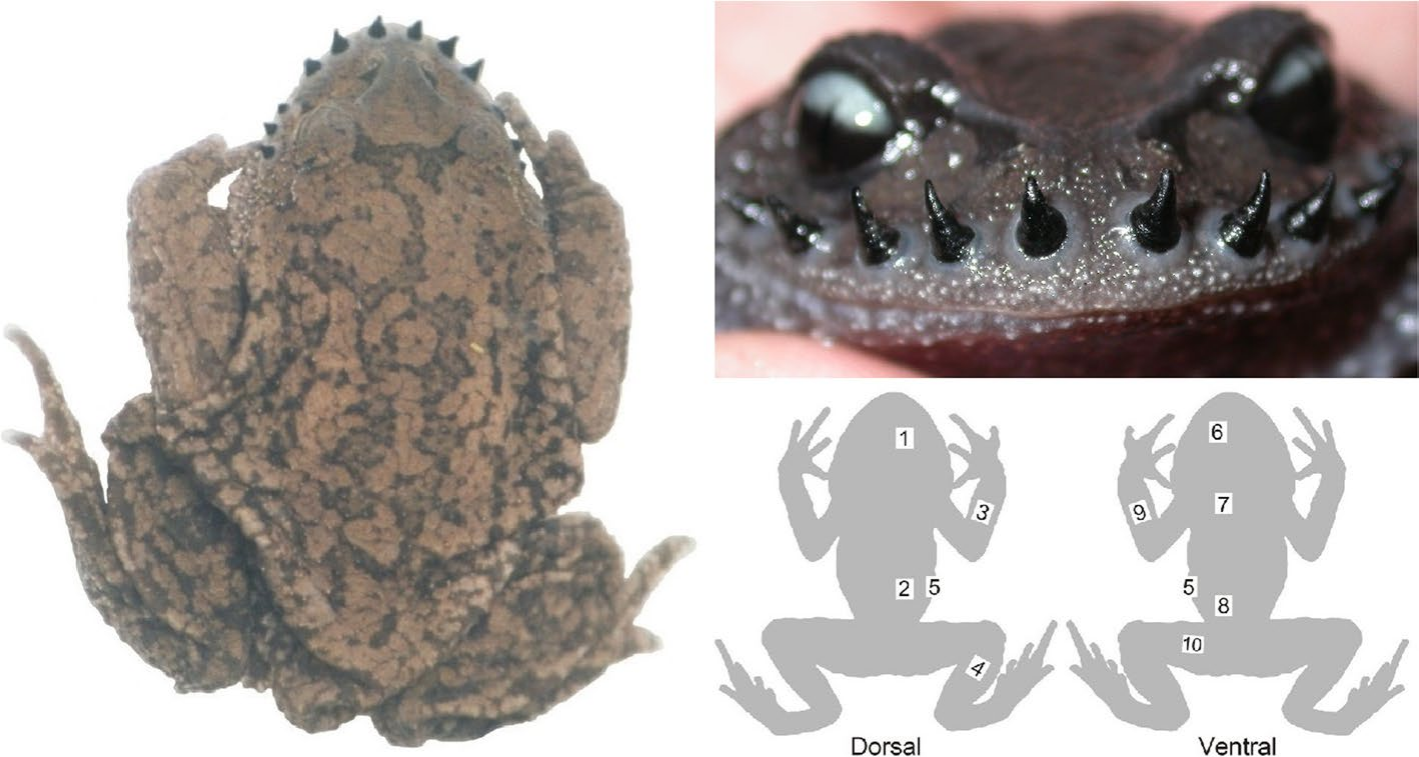
Two male nuptial traits in *Leptobrachium boringii*, maxillary spines and loose skin, and ten sampling locations of skin sections. 1: dorsal head; 2: back; 3: dorsal forearm; 4: dorsal distal hindlimb; 5: lateral abdomen; 6: ventral head; 7: chest; 8: posterior belly; 9: ventral forearm; 10: ventral proximal hindlimb

The Emei mustache toad, *Leptobrachium boringii* (Liu, 1945), provides an ideal system for such exploration. This subtropical East Asian megophryid inhabits mountain valleys at elevations of 600–1,700 m a.s.l., breeds in early spring from February to March in permanent streams, and remains terrestrial in broad-leaved forests during the nonbreeding season [16, 17]. It exhibits male-biased sexual size dimorphism, with, for example, males reaching a snout-vent length (SVL) of 69.8–89.0 mm and females ranging from 58.6–76.0 mm [18]. Male *L. boringii* entering the breeding stream bear nuptial traits, including conspicuously enlarged arms, loose skin with an increased respiratory surface area, and 10–16 maxillary keratinized spines (Fig. 1). They construct submerged nests under rocks, emit advertisement calls from the nest, compete in choruses, and engage in underwater intrasexual combat for nest ownership [19–21]. The spines are used to stab the opponent, which can be steadied on the spines by the forelimbs, often causing many red spot-like hemorrhages and occasionally a puncture in the skin [19, 20]. In contrast, breeding females have no such nuptial traits and probably have less intense underwater behaviors. The female performs tactile inspection of a potential mate’s nest, drives rotation of the pair in amplexus while producing a doughnut-shaped egg mass, and departs the breeding nest after oviposition, leaving the male to provide paternal care and attract additional mates [20–23]. One may further expect seasonal changes or sex differences in the extent of skin vascularization [3, 24]. As expected, in our preliminary prospection, skin histological sections showed capillaries penetrating the epidermis.

To explore possible relationships between the capillary penetration extent and other skin characters, it is reasonable to consider characters that affect respiration and protection, such as subepidermal capillary density and epidermal thickness. With shared effects, the capillary penetration extent may covary with these characters. In amphibians, such skin characters usually vary significantly in different body regions and between dorsal and ventral sides [1, 7, 25–28]. Using *L. boringii* as an example, these relationships can be explored by performing relation analysis of data from diverse body regions [7].

**Fig. 2:**
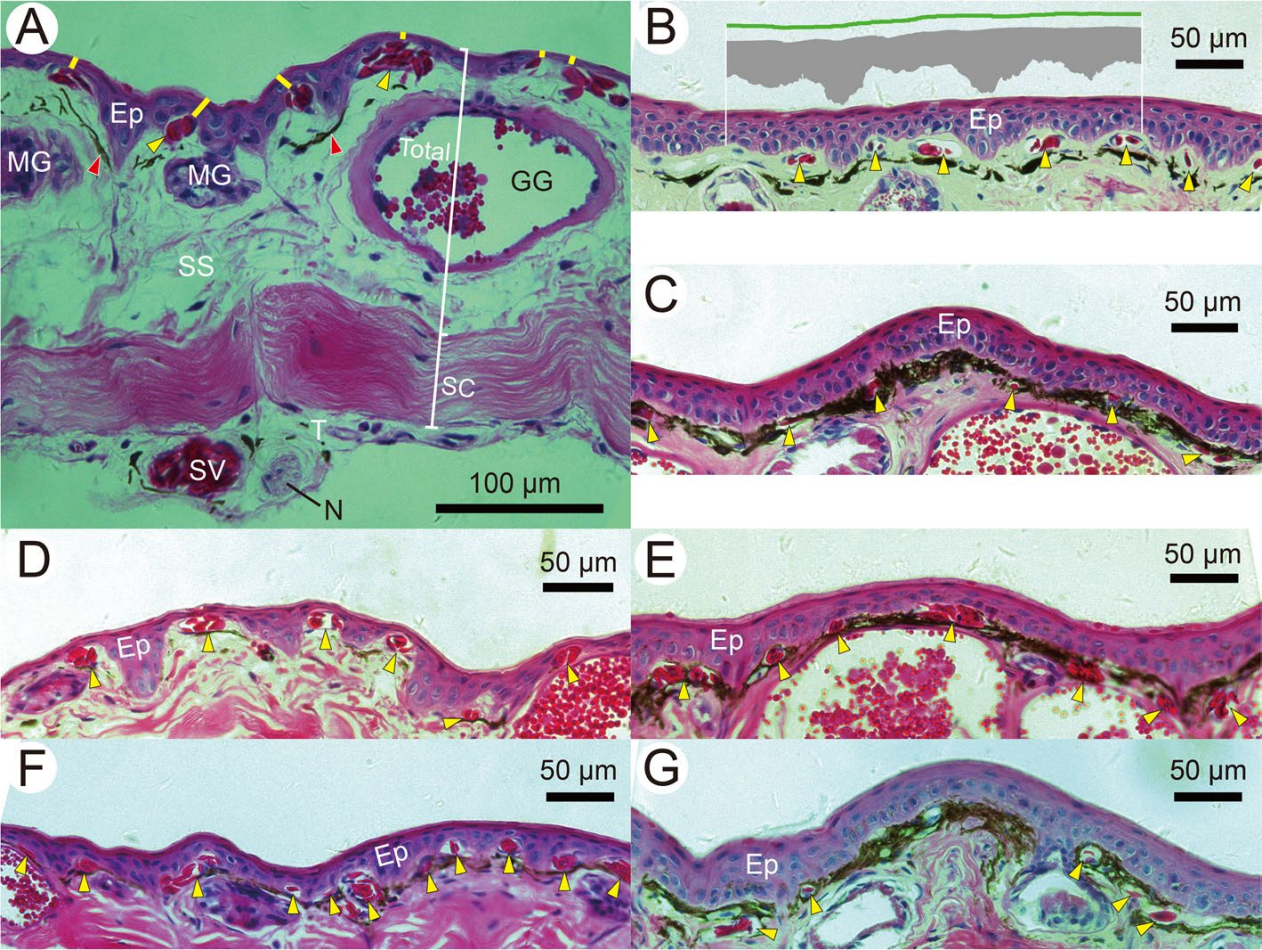
Micrographs exemplifying the general organization and measurements of the skin and varying extents of the penetration of capillaries into the epidermis of *Leptobrachium boringii*: (**A**) lateral abdominal skin of a breeding male with a snout-vent length (SVL) of 79.4 mm; (**B**) dorsal head and (**C**) ventral proximal hindlimb skin of a nonbreeding male with an SVL of 80.6 mm; (**D**) dorsal head and (**E**) ventral proximal hindlimb skin of a breeding male with an SVL of 74.7 mm; and (**F**) dorsal head and (**G**) ventral proximal hindlimb skin of a breeding female with an SVL of 71.2 mm. Each yellow line approximates the minimum diffusion path of a capillary, yellow arrows indicate subepidermal capillaries, and red arrows indicate melanophores. The green line and the gray area show sections of a freehand line along and a polygon matching the epidermis, respectively. Ep: epidermis; GG: granular gland; MG: mucous gland; N: nerve plexus; SC: *stratum compactum* thickness; SS: *stratum spongiosum*; SV: subcutaneous vessel; Total: total skin thickness; T: tela subcutanea. All from the right side of the body

More unusually, underwater fighting with spines in this species implies two strong conflicting and simultaneous selection pressures on epidermal thickness: thin for effective cutaneous respiration required by metabolically costly combat but thick for protection against sharp spines. Combat typically lasts 2–5 min [19, 21], and two males have been observed to fight five rounds within 50 min, with only one male occasionally holding its nostrils briefly out of the water in the intervals between fights [20]. Once caused by stabbing, even tiny open wounds with broken subepidermal capillaries may further disadvantage the animal by chemically attracting leeches [29]. On the other hand, selection for a thick epidermis in breeding males may be reduced by the increased structural extensibility of their loose skin, which must be stretched before being punctured [30]. The significance of this effect of skin looseness on amphibian life history remains unknown. In *L. boringii* and assuming that a thick epidermis with little penetration of capillaries confers protection against stabbing, breeding males can be compared with nonbreeding males that exhibit no spines or loose skin and presumably do not fight. Another character suitable for such comparison is the thickness of the *stratum compactum*, the lower dermal layer composed mainly of compact collagen fibers (Fig. 2A). This layer provides substantial mechanical strength, and its large thickness can be assumed to confer resistance to puncture [31, 32]. If similar or less favorable conditions are found in breeding males, the results can be explained by skin looseness providing an advantage in protection against stabbing during combat.

Intraspecific comparisons of *L. boringii* may further facilitate a more comprehensive understanding of the role of erythrocyte size in the enhancement of respiration in amphibians. Smaller red blood cells have higher surface/volume ratios, allowing for more efficient gas exchange, and some amphibian species have been reported to reduce their red blood cell size to augment respiration [33, 34]. This strategy may have little impact on their cutaneous oxygen exchange, which is likely diffusion limited because in contrast to the respiratory membranes of lungs and gills, the skin epidermis is too thick for rapid transfer of oxygen [1, 12, 35]. Taking advantage of the fact that red blood cells are often washed out of large subcutaneous vessels as single cells and found lying on slides containing skin sections, erythrocyte size proxies such as area and length [36–39] can be compared between breeding and nonbreeding males.

Based on samples from different breeding stages and sexes and on paraffin sections of 10 skin regions, this study uses *L. boringii* as an example to quantify the penetration extent of capillaries into the epidermis, explore associations between the extent and other variables, integrate the extent into the examination of a defensive role of skin looseness, and assess whether a seasonal shift to high levels of skin breathing involves a reduction in red blood cell size.

## Methods

### Sampling and paraffin skin sections

Nine adult *L. boringii* specimens were collected at Mt. Emei (N29.6°, E103.4°): three breeding males in February, CIB-IOZCAS08143 (SVL 74.7 mm), 10369 (80.3), and 10370 (79.4); three nonbreeding males in May to July, 08867 (69.5), 09432 (80.6), and 09433 (72.2); and three breeding females in February, 08148 (70.3), 08150 (72.8), and 08151 (71.2). Within 20 h after capture, the frogs were anesthetized and sacrificed by immersion in an ethanol solution of a slowly increasing but less than ~ 5% concentration, i.e., a small amount of 95% ethanol was added at the container edge distant to the frog around 10 times. Then, they were fixed in 8% formalin, placed in flowing water for 24 h, and stored in 70% ethanol before examination.

For each specimen, skin fragments approximately 6 × 5 mm in size were sampled from 10 regions across the right side of the body, including the dorsal head, back, dorsal forearm, dorsal distal hindlimb, lateral abdomen, ventral head, chest, posterior belly, ventral forearm, and ventral proximal hindlimb. This allowed the preservation of all skin on one side of the body of the specimen, and it was relatively convenient to conduct the sampling in these regions. Their locations are shown in Fig. 1. The obtained fragments were dehydrated with ascending concentrations of ethanol, cleared with xylene, and embedded in paraffin with a melting point of 52–54 °C. Cross-sections 6 μm thick were made using a Leica RM2025 microtome, stained with hematoxylin and eosin, and sealed with neutral balsam. The two types of integumentary skeletons found in anurans, osteoderm and lamina calcarea, can be detected by hematoxylin and eosin staining if present [40–42].

### Data extraction

The sections were observed and photographed using a Leica DM2500 light microscope equipped with a Leica DFC495 charge-coupled device. For each skin fragment, 30 micrographs were obtained under a Leica 10 × objective lens (HC PL Fluotar, numerical aperture 0.30) from five section sets separated by approximately 600-μm intervals, with each set containing six sections at approximately 36-μm intervals on a single slide. These micrographs were analyzed with ImageJ version 1.52a [15] to quantify the minimum distance from a capillary cavity to the epidermal surface (i.e., minimum diffusion path length), epidermal thickness, capillary density, amount of red blood cells per capillary, thickness of the *stratum compactum*, and total skin thickness (Fig. 2A). To estimate the epidermal thickness of a micrograph, the area of the epidermis was divided by the average length of three replicated freehand lines drawn along the epidermis (Fig. 2B) [13, 14]. Across the 30 micrographs, the extent of the penetration of capillaries into the epidermis was calculated as the ratio of the average of individual minimum diffusion path lengths to the average epidermal thickness. As a measure of perfused capillary density, the number of subepidermal capillaries in a micrograph was divided by the average freehand line length [43–46]. Given the uncertainty in counting red blood cells in micrographs, four grades of blood cell numbers per capillary were defined: one, two or three, four to nine, and 10 or more cells. In addition, a simplified three-grade classification based on cell number was also applied: one, two or three, and four or more. The total thickness and *stratum compactum* thickness were measured once for each micrograph, in most cases along a straight line crossing the epidermal surface at a point where one straight line marked on the monitor screen crossed the surface. If the corresponding *stratum compactum* layer was broken, the closest suitable part was used. In a few micrographs, a suitable part was not available, and no measurements were made. When such missing data occurred, a mean value was calculated for each section set to obtain the regional average.

Micrographs were also obtained under a Leica 63 × objective lens (HCX APO, water immersion, numerical aperture 0.90) and analyzed with ImageJ to measure *stratum corneum* thickness and red blood cell size proxies. For each skin fragment, 10 *stratum corneum* thickness measurements were made across one section. The areas, lengths, and widths of 50 red blood cells were measured for each frog, and the length to width ratios of these cells were calculated.

### Comparison between breeding stages and sexes

Generalized linear mixed models (GLMMs) were used to test for differences in the extent of the penetration of capillaries into the epidermis, minimum diffusion path length, epidermal thickness, density of perfused subepidermal capillaries, *stratum compactum* thickness, total skin thickness, red blood cell area, red blood cell length and width, and the length/width ratio among breeding male, nonbreeding male, and breeding female groups. A cumulative link mixed model (CLMM) was fitted to test for differences in the grade of red blood cell count per subepidermal capillary among groups. The tests were performed in R [47], including body region and SVL as fixed effects [1, 27, 28] and individual as a random effect. The GLMMs were implemented using the lmer function of the lme4 package, and the CLMM was fitted using the clmm function in the ordinal package. For pairwise comparisons between groups, the glht function in the multcomp package and the emmeans function of the emmeans package were used with GLMMs and CLMMs, respectively.

For the density of perfused subepidermal capillaries, across the 10 sampled body regions, a correlation between the group mean of nonbreeding males and the difference in breeding male and nonbreeding male group means was tested for with Pearson’s method in SPSS version 12.0. The normality of these two variables was tested and confirmed by Kolmogorov‒Smirnov tests conducted in SPSS (both *P* > 0.05).

### Exploring relationships between variables

For each group, GLMMs were fit using lmer in lme4 to examine associations between the variables across the 10 body regions, based on regional averages for each frog and including individual as a random effect. Four variables of the epidermis and subepidermal capillaries were considered: the penetration extent of capillaries, minimum diffusion path length, epidermal thickness, and perfused capillary density. This resulted in six combinations. Possible associations between *stratum compactum* thickness and epidermal thickness or capillary penetration extent were also evaluated. The marginal coefficient of determination (*R*^2^m) and conditional coefficient of determination (*R*^2^c) were obtained using the r.squaredGLMM function of the R package MuMIn.

## Results

### Comparisons between breeding stages and sexes

**Fig. 3:**
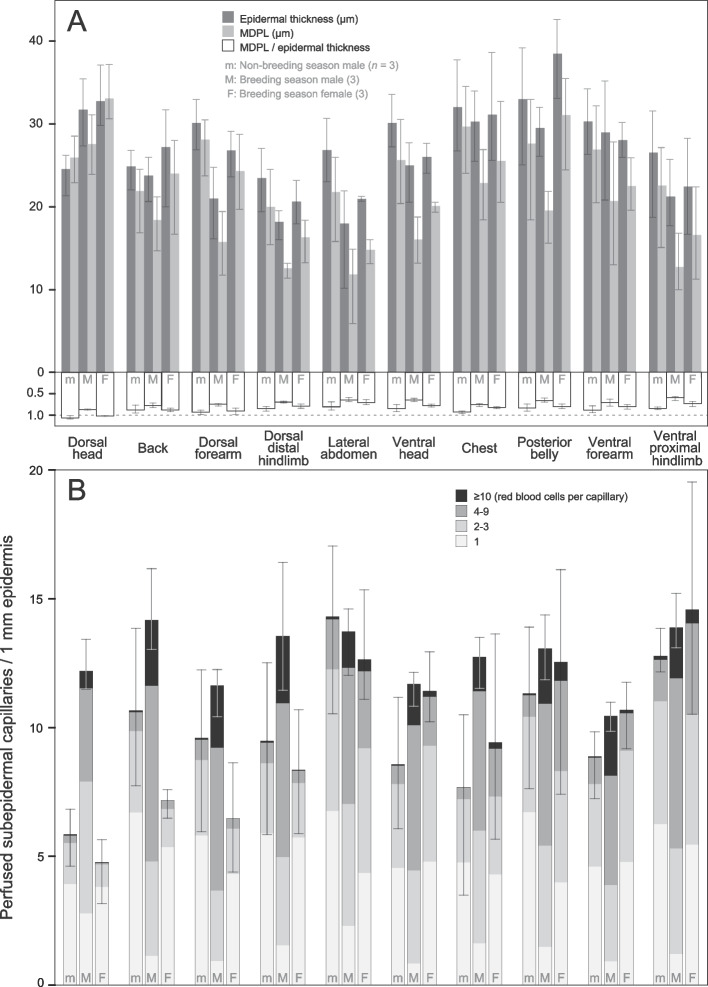
Variation in epidermal thickness and penetration of capillaries into the epidermis (**A**) and vascularity beneath the epidermis (**B**). Bars and vertical lines are means and ranges of each individual’s mean values, respectively. MDPL: average minimum diffusion path length of subepidermal capillaries. Note that there is some uncertainty in counting red blood cells in micrographs, especially for capillaries containing several or more cells, and that combining the grades of 4–9 and ≥ 10 cells result in the same pattern

With similar epidermal thicknesses, more intense penetration of capillaries into the epidermis was found in the breeding male group than in the nonbreeding male and breeding female groups (Figs. 2 and 3). No osteoderm or lamina calcarea was found in their skin. Typically, the epidermis of these frogs was composed of three to six cell layers, including an outermost *stratum corneum* 1.5–3.5 μm thick and one or two adjacent layers of flattened cells. In the GLMMs fitted using raw values for individual micrographs or regional averages, group and SVL had no effect (all *P* > 0.05) and body region showed a significant effect (both *P* < 0.001) on epidermal thickness. Among body regions, the group means of epidermal thickness for breeding males, nonbreeding males, and breeding females ranged from 18–32, 23–33, and 21–38 μm, respectively. Based on raw values and considering each region alone, group and SVL again showed no effect on epidermal thickness (all *P* > 0.05). Both group (*P* = 0.006) and body region (*P* < 0.001) had a significant effect on the capillary penetration extent, while SVL did not (*P* > 0.05). The minimum diffusion path length to epidermal thickness ratio of breeding males was significantly lower than those of nonbreeding males and breeding females (both *P* < 0.001), which were not significantly different from each other (*P* > 0.05) (Table 1).

**Table 1:** Skin and erythrocyte variables expressed as mean ± SD for the three groups

Variable	Breeding male	Non-breeding male	Breeding female
Capillary penetration extent	0.71 ± 0.09	0.88 ± 0.09	0.82 ± 0.10
CD (dorsal head; capillaries/mm)	12.2 ± 2.6	5.8 ± 2.7	4.8 ± 2.5
MDPL (dorsal forearm; μm)	16 ± 9	29 ± 8	23 ± 8
MDPL (dorsal distal hindlimb; μm)	13 ± 8	21 ± 8	16 ± 7
Erythrocyte area (μm^2^)	180 ± 24	150 ± 17	178 ± 30
Erythrocyte length (μm)	21 ± 2	19 ± 1	20 ± 2
Erythrocyte width (μm)	11 ± 1	10 ± 1	11 ± 1
Erythrocyte length/width ratio	2.0 ± 0.2	1.9 ± 0.2	1.9 ± 0.2
*Stratum compactum* thickness (μm)	56 ± 19	66 ± 18	47 ± 15
Total skin thickness (μm)	174 ± 54	187 ± 50	155 ± 56

Capillary penetration extent: ratio of the average minimum diffusion path length of perfused subepidermal capillaries (MDPL) to the average epidermal thickness; CD: density of perfused subepidermal capillaries. The skin variable values were based on the 10 sampled body regions if not specified

**Fig. 4:**
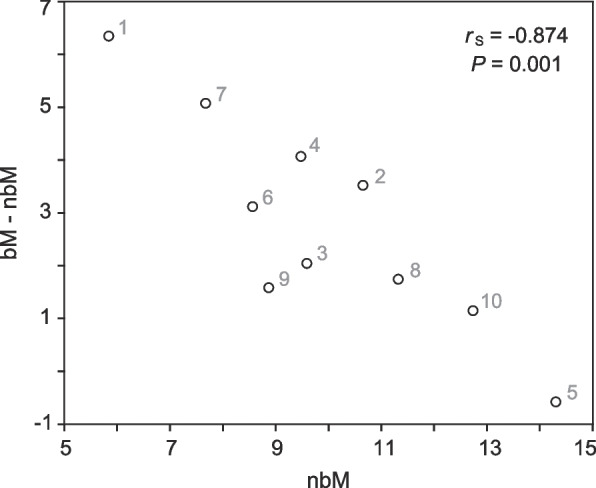
Negative correlation between the regional average densities of perfused subepidermal capillaries for nonbreeding males (nbM) and the differences in such densities between breeding and nonbreeding males (bM − nbM). *n* = 3 for both seasons. *r*_S_: Pearson correlation coefficient. Unit: capillaries per millimeter of epidermis. 1: dorsal head; 2: back; 3: dorsal forearm; 4: dorsal distal hindlimb; 5: lateral abdomen; 6: ventral head; 7: chest; 8: posterior belly; 9: ventral forearm; 10: ventral proximal hindlimb

For three body regions, a statistically significantly higher density or shorter minimum diffusion path length of perfused subepidermal capillaries was found in breeding males than in nonbreeding males and breeding females. For most sampled body regions, breeding males had a larger group mean value of capillary density and a smaller group mean value of minimum diffusion path length than the other two groups (Fig. 3). The GLMM analyses based on raw values or regional averages showed a significant effect of body region (both *P* < 0.001) and no effect of group or SVL (all *P* > 0.05) on both the density and minimum diffusion path length variables. The group means of the two variables for each body region ranged from 10.4–14.2, 5.8–14.3, and 4.8–14.6 capillaries/mm and 12–28, 20–30, and 15–33 μm in breeding males, nonbreeding males, and breeding females, respectively. For the density variable, the difference in group means between breeding and nonbreeding males was strongly negatively correlated with the group mean of nonbreeding males across body regions (Pearson correlation coefficient –0.874, *P* = 0.001) (Fig. 4). When each body region was individually analyzed using raw values, group had a significant effect on the density variable in the dorsal head (*P* = 0.003) and on the path length variable in the dorsal forearm (*P* = 0.037) and dorsal distal hindlimb (*P* = 0.034) (Table 1), and SVL had no effect on either variable (all *P* > 0.05). In the dorsal head region, the perfused subepidermal capillary density of breeding males was higher than those of nonbreeding males and breeding females (both *P* < 0.001), which were not significantly different from each other (*P* > 0.05). For the dorsal forearm, the minimum diffusion path length of breeding males was significantly shorter than those of nonbreeding males (*P* < 0.001) and breeding females (P = 0.030), which were not significantly different from each other (*P* > 0.05). Similarly, for the dorsal distal hindlimb, the path length of breeding males was shorter than those of nonbreeding males (*P* < 0.001) and breeding females (*P* = 0.042), which were not significantly different from each other (*P* > 0.05).

With similar size proxies and length/width ratios of red blood cells, breeding males showed a higher erythrocyte content of subepidermal capillaries than the other two groups (Fig. 3B, Table 1). The GLMMs showed no effect of group or SVL on red blood cell area, length, width, or length/width ratio (all *P* > 0.05). The CLMM based on raw values indicated significant effects of SVL (*P* < 0.001), body region, and group on erythrocyte content classified into either three or four grades. In both cases, the posterior belly region was significantly different from five regions (all *P* < 0.001) but not from the same other four regions (all *P* > 0.05), and the erythrocyte content of subepidermal capillaries in breeding males (mostly two or more cells) was higher than those in the other two groups (mostly one; both *P* < 0.001), which were not significantly different from each other (*P* > 0.05).

The *stratum compactum* thickness was found to be greater in nonbreeding males than in the other two groups, and no overall difference in total skin thickness was found between groups (Table 1). The GLMM based on raw values showed significant effects of group (*P* = 0.013), body region (*P* < 0.001), and SVL (*P* = 0.049) on the thickness of the *stratum compactum*. For this variable, nonbreeding males had values larger than those of breeding males and breeding females (both *P* < 0.001), which were not significantly different from each other (*P* > 0.05). The GLMM based on raw values indicated a significant effect of body region (*P* < 0.001) and no effect of group or SVL (both *P* > 0.05) on the total thickness.

### Relationships between variables

**Fig. 5:**
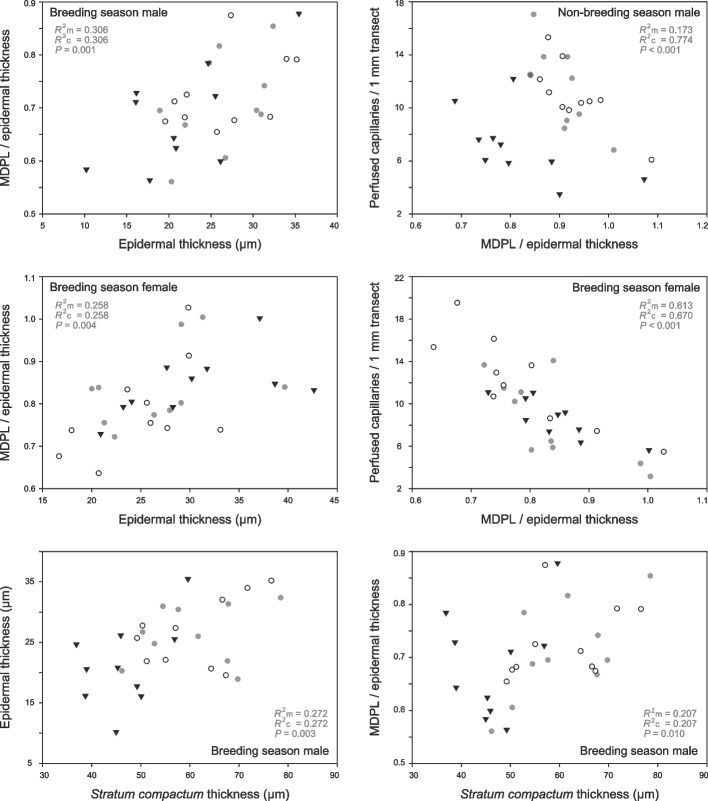
Correlations between the extent of capillaries penetrating the epidermis, thickness of the epidermis, perfused subepidermal capillary density, and *stratum compactum* thickness across 10 body regions in *Leptobrachium boringii*. Symbols are used to distinguish individuals of the same group, and regional mean values are plotted. The marginal (*R*^2^m) and conditional (*R*^2^c) coefficients of determination and *P* values were derived from generalized linear mixed models where individual was included as a random factor. MDPL: average minimum diffusion path length of subepidermal capillaries

Correlations between the penetration extent of capillaries, average minimum diffusion path length of perfused subepidermal capillaries, and epidermal thickness were identified by GLMM analyses (Table 2). The minimum diffusion path length to epidermal thickness ratios were lower in body regions with smaller epidermal thicknesses in breeding males and breeding females (Fig. 5) but not in nonbreeding males (*P* > 0.05) (Fig. S1). In all three groups, the ratios were lower in body regions with shorter minimum diffusion path lengths. The minimum diffusion path length was closely and positively correlated with epidermal thickness in each group.

**Table 2:** Correlations between four skin variables across 10 body regions in *Leptobrachium boringii*

Variable pair	Breeding male	Non-breeding male	Breeding female
CPR ~ EP [+]	0.306 / 0.306 / *P* = 0.001	0.002 / 0.346 / *P* = 0.810	0.258 / 0.258 / *P* = 0.004
CPR ~ MDPL [+]	0.615 / 0.615 / *P* < 0.001	0.364 / 0.373 / *P* = 0.010	0.579 / 0.579 / *P* < 0.001
MDPL ~ EP [+]	0.890 / 0.890 / *P* < 0.001	0.728 / 0.830 / *P* < 0.001	0.883 / 0.883 / *P* < 0.001
CD ~ EP	0.061 / 0.061 / *P* = 0.179	0.001 / 0.431 / *P* = 0.866	0.055 / 0.185 / *P* = 0.190
CD ~ MDPL [-]	0.072 / 0.072 / *P* = 0.145	0.111 / 0.740 / *P* = 0.026	0.225 / 0.309 / *P* = 0.006
CD ~ CPR [-]	0.058 / 0.058 / *P* = 0.192	0.173 / 0.774 / *P* < 0.001	0.613 / 0.670 / *P* < 0.001

The marginal (left) and conditional (middle) coefficients of determination and *P* values were derived from generalized linear mixed models where individual was included as a random factor. EP: epidermal thickness; MDPL: average minimum diffusion path length of perfused subepidermal capillaries; CPR: capillary penetration ratio (MDPL/EP); CD: density of perfused subepidermal capillaries; [+]: positive correlation; [-]: negative correlation

Perfused subepidermal capillary density was not correlated with epidermal thickness in any group or with either capillary penetration extent (Fig. S1) or minimum diffusion path length in breeding males (all *P* > 0.05) (Table 2). The densities were significantly higher for body regions with shorter minimum diffusion path lengths in nonbreeding males and breeding females. The density was also negatively correlated with the minimum diffusion path length to epidermal thickness ratio in nonbreeding males and breeding females (Fig. 5).

On the other hand, epidermal thickness and capillary penetration extent were correlated with *stratum compactum* thickness in breeding males (Fig. 5) but not in the other two groups (all *P* > 0.05) (Fig. S1). In breeding males, epidermal thicknesses were lower in body regions with thinner *stratum compactum* thicknesses (*R*^2^m 0.272, *R*^2^c 0.272; *P* = 0.003), and the minimum diffusion path length to epidermal thickness ratios were smaller in such regions (0.207, 0.207; *P* = 0.010).

## Discussion

### Penetration extent of capillaries into the epidermis

The quantified capillary penetration extent not only shows statistically significant associations with other variables but can also be more sensitive in revealing structural differences in the skin than other variables. In the Mt. Emei population of *L. boringii*, lower ratios of minimum diffusion path length to epidermal thickness, i.e., greater extents, may be found in body regions with smaller epidermal thicknesses, shorter minimum diffusion path lengths, higher perfused subepidermal capillary densities, or smaller *stratum compactum* thicknesses (Fig. 5). Among the four structural variables of the epidermis and subepidermal capillaries, only capillary penetration extent helped to identify two combinations of the three groups of individuals. It correlated with epidermal thickness in breeding males and females but not in terrestrial nonbreeding males, and it correlated with perfused capillary density in breeding females and nonbreeding males but not in breeding males, which exhibit intense underwater behaviors [19–21]. In contrast, each of the epidermal thickness, minimum diffusion path length, and perfused capillary density variables contributed to identifying one of the two combinations. Moreover, among these four variables, capillary penetration extent was also the only one showing overall differences between groups in the GLMM. It was greater in breeding males. This feature of the trait may be partly attributed to its generally lower intragroup variation (mean relative SD = 6.6%) than in the other three variables (15.4 to 20.8%) in individual body regions (Fig. 3).

The detected associations can be informative and useful. Considering penetration as a trade-off between promoting cutaneous respiration and maintaining overall epidermal thickness [1, 12], one may expect a great capillary penetration extent where the epidermis can be thin in skin-breathers. Indeed, the capillary penetration extent was negatively correlated with epidermal thickness in aquatic breeding males and females that rely on skin breathing but not in terrestrial nonbreeding males. This result further implies that such structural remodeling for augmenting skin respiration also exists in females. On the other hand, unlike breeding males, female Emei mustache toads do not have conspicuously loose skin, which increases respiratory surface area and occurs in a variety of amphibians [1, 27, 48–51]. Therefore, it seems reasonable to speculate that the feature of thinner epidermis with greater penetration perhaps develops relatively easily and is widespread among skin-breathing amphibians. In the following discussion, we will present another example of the application of the detected associations. Awareness of the usefulness of the capillary penetration ratio may inspire new methods for quantifying the penetration.

### Effect of skin looseness in combat

Our histological results show no sign of cutaneous structural reinforcement against stabbing in breeding males of *L. boringii*, which fight each other using maxillary nuptial spines underwater. The samples lack possibly protective integumentary skeletons [40, 52], and they do not have particularly thick epidermis or *stratum compactum* (largely 20–40 and 40–70 μm, respectively) when compared to those of other frogs of similar or smaller body sizes [8, 28, 53–55]. Stabbing can cause open wounds that chemically attract leeches [19, 29], which often parasitize and even kill adult amphibians [56–60]. In breeding males of *L. boringii*, a few to dozens of unidentified leeches several millimeters in length have frequently been found on submerged individuals bearing red spot-like hemorrhages in the skin from stabbing (Y. Zheng, personal observation). It is likely that these individuals had small open wounds as well. Compared with nonbreeding males, however, breeding males had a similarly thick epidermis but more intense penetration of capillaries (Fig. 3) and had a thinner *stratum compactum*, i.e., less favorable conditions for resistance to puncture. Nevertheless, unlike in nonbreeding males, epidermal thickness, *stratum compactum* thickness, and the ratio of minimum diffusion path length to epidermal thickness were positively correlated with each other across body regions in breeding males (Fig. 5), implying that they covaried with selective pressure from stabbing. These findings can be explained by the increased extensibility of the breeding male’s loose skin conferring an advantage against stabbing because the skin has to be stretched before being punctured [30]. Related to the engineering design of exchange surfaces, this implies a case of looseness contributing to both increasing the surface area and preventing damage.

In amphibians, a damage-preventing role of skin looseness remains to be tested by direct quantitative evidence. Beyond this role, it is possible, for instance, that by increasing the freedom of movement, skin looseness enables males of the common frog *Rana temporaria* to struggle in a ‘mating ball’ consisting of several males trying to clasp a female [51, 61].

### Erythrocyte size and subepidermal capillary erythrocyte content

We did not find evidence of reduced red blood cell size in breeding males of *L. boringii*. Breeding males, nonbreeding males, and breeding females had red blood cells with similar size proxies and length/width ratios, and their red blood cell area (mean = 169 μm^2^) or length x width (20 × 10 μm) is not particularly small when compared with that of other frog species [33, 38, 62]. A high surface/volume ratio of small red blood cells benefits gas exchange, and some amphibians have been reported to reduce red blood cell size to respond to hypoxia [33, 34, 63]. However, since cutaneous oxygen exchange is primarily limited by diffusion rate, not by the rate of oxygen binding to hemoglobin [12, 35, 64], a transformation for achieving high levels of cutaneous respiration likely will not involve a reduction in red blood cell size in the life history of amphibians. The present study provides early evidence from a natural population that supports our speculation. Further intraspecific comparisons may be made in obligate skin-breathers exhibiting seasonal metabolically demanding behaviors.

Despite similar erythrocyte sizes, the subepidermal capillaries of breeding male *L. boringii* can have a considerably higher erythrocyte content than those of nonbreeding males and breeding females regardless of capillary density (Fig. 3). However, as this study was not designed to compare erythrocyte content, such a difference should probably only be used for hypothesis generation. A high erythrocyte content indicates a high hemoglobin content, which facilitates maintaining a low blood oxygen partial pressure (PO_2_) that increases with percent hemoglobin oxygen saturation [65, 66]. In *L. boringii*, breeding males are unique in having underwater behaviors that require high levels of skin breathing. We therefore hypothesize that by facilitating the maintenance of steep PO_2_ gradients between the environment and blood, a great lineal density of hemoglobin along subepidermal capillaries can increase cutaneous oxygen uptake. This remains to be tested.

### Subepidermal capillary density

Our lineal density estimates of perfused subepidermal capillaries are informative regarding whether the skin of *L. boringii* is well vascularized compared to that of other amphibians. A micrograph showing the subepidermal capillary network can be utilized to link the lineal density of capillaries and the commonly used capillary mesh density, of which the latter ranges from approximately 20–300 meshes per mm^2^ of skin in the amphibians examined [1, 7, 25, 53, 67–69]. Jasiński & Miodoński [70] presented such micrographs for the dorsal skin of the hybridogenetic water frog *Pelophylax esculentus*. Their Figs. 4–6 showed three micrographs with areal densities of approximately 190, 175, and 145 capillary meshes per mm^2^ and, averaged across the four edges of each micrograph, lineal densities of 18.1, 17.3, and 13.6 capillaries per mm, respectively. The last value is comparable to our estimates from breeding males, with an average of 10.4–14.2 perfused capillaries per mm for various body regions (Fig. 3). Therefore, given the possible presence of nonperfused capillaries, in amphibians, breeding male *L. boringii* likely have at least a moderate density of skin subepidermal capillaries.

For the same reason, the negative correlation across body regions between the perfused capillary density of nonbreeding males and its difference from that of breeding males (Fig. 4) should be interpreted with caution. Skin capillary density often varies considerably among body regions in anurans [7]. In response to environmental hypoxia, an increase in cutaneous capillary density and hence functional respiratory surface area has been found in larval bullfrogs (*Lithobates catesbeianus*) [67]. The correlation implies at most that if there is a seasonal increase in subepidermal capillary density in the skin of male *L. boringii*, it is significant mainly in the originally less vascularized skin regions.

### Exclusive skin breathing and metabolically demanding behaviors

Evidence from the Emei mustache toad supports the possibility that skin-breathing amphibians are not necessarily limited to low activity. In amphibians, cutaneous respiration has generally been regarded as inadequate and of primary value in adults with relatively low metabolic demands [1, 27, 71, 72]. Previous observations indicated that during exclusive skin breathing, breeding male Emei mustache toads are capable of engaging in several rounds of intense combat lasting a few minutes each within 1 h [19–21]. In addition to the conspicuous increase in skin looseness and hence respiratory surface area [73], our histological results are also consistent with a high cutaneous gas exchange capacity of these toads. The skin of amphibians is in general heavily vascularized with respect to gas exchange [1]. As mentioned above, samples of breeding males probably had at least moderately dense subepidermal capillaries compared to those of other amphibians. After regional shortening, the average distance from these capillaries to the skin surface ranged from 12–20 μm in seven of the 10 body regions (Fig. 3). This diffusion distance is relatively short and close to the smallest records of approximately 4–10 μm for amphibians [1, 7, 12, 53, 55, 74–77]. On the other hand, fighting with spines may be energy-efficient in harming the opponent and hence to some extent make the combat affordable for high levels of cutaneous respiration. Underwater combat with pointed weapons also occurs in breeding males of the hellbender salamander *Cryptobranchus alleganiensis* (biting) and probably the hairy frog *Astylosternus robustus* (claws), which are regarded to exhibit a high cutaneous respiration capacity aided by well-vascularized skin folds or projections, respectively [3, 72, 78–83].

Another behavior that can require efficient skin breathing is acoustically competing in underwater choruses. In some alsodid, megophryid, pelobatid, ranid, and telmatobiid frogs, the cooccurrence of loose skin and underwater calling has been noted and interpreted as the performance of the latter benefitting from a high respiratory surface-to-volume ratio [84, 85]. In amphibians, skin folds with dense capillaries often develop to facilitate skin breathing [1, 27], but whether the loose skin of these frogs is well vascularized remains mostly unknown. As an exception, the baggy skin of the Lake Titicaca frog, *Telmatobius coleus*, has clearly been shown to exhibit dense capillaries penetrating extensively into the epidermis [6, 7, 72, 76, 86]. This study suggests that the loose skin of breeding male *L. boringii* is also well vascularized close to the surface.

## Conclusions

This study provides the first procedure to quantify the penetration extent of capillaries into the epidermis. The minimum diffusion path length/epidermal thickness ratio can serve as a useful metric in, for example, collecting early evidence consistent with skin looseness contributing to defense during combat in amphibians. This may attract interest for applications and developing methods to quantify penetration.

### Supplementary Information

**Additional file 1: Figure S1. **Plots between the extent of capillaries penetrating the epidermis, thickness of the epidermis, perfused subepidermal capillary density, and *stratum compactum* thickness across ten body regions in *Leptobrachium boringii*. Symbols are used to distinguish individuals of the same group, and regional mean values are plotted. The *P* values were derived from generalized linear mixed models where individual was included as a random factor. MDPL: average minimum diffusion path length of subepidermal capillaries.

## Data Availability

The datasets analyzed are available on ScienceDB (10.57760/sciencedb.08988).

## Abbreviations

SVL: Snout-vent length
GLMM: Generalized linear mixed model
CLMM: Cumulative link mixed model
*R*^2^m: Marginal coefficient of determination
*R*^2^c: Conditional coefficient of determination
PO_2_: Oxygen partial pressure
