# Supplementary material for: Male spiny frogs enter the underwater battlefield with loose skin exhibiting enhanced penetration of capillaries into the epidermis
Source: Zoological Lett. 2023 Oct 6;9:19. doi: 10.1186/s40851-023-00219-4 (PMC10557191; doi:10.1186/s40851-023-00219-4)
Supplement: Supplementary file 1 — Additional file 1: Figure S1. Plots between the extent of capillaries penetrating the epidermis, thickness of the epidermis, perfused subepidermal capillary density, and stratum compactum thickness across ten body regions in Leptobrachium boringii. Symbols are used to distinguish individuals of the same group, and regional mean values are plotted. The P values were derived from generalized linear mixed models where individual was included as a random factor. MDPL: average minimum diffusion path length of subepidermal capillaries. [file 40851_2023_219_MOESM1_ESM.pdf]

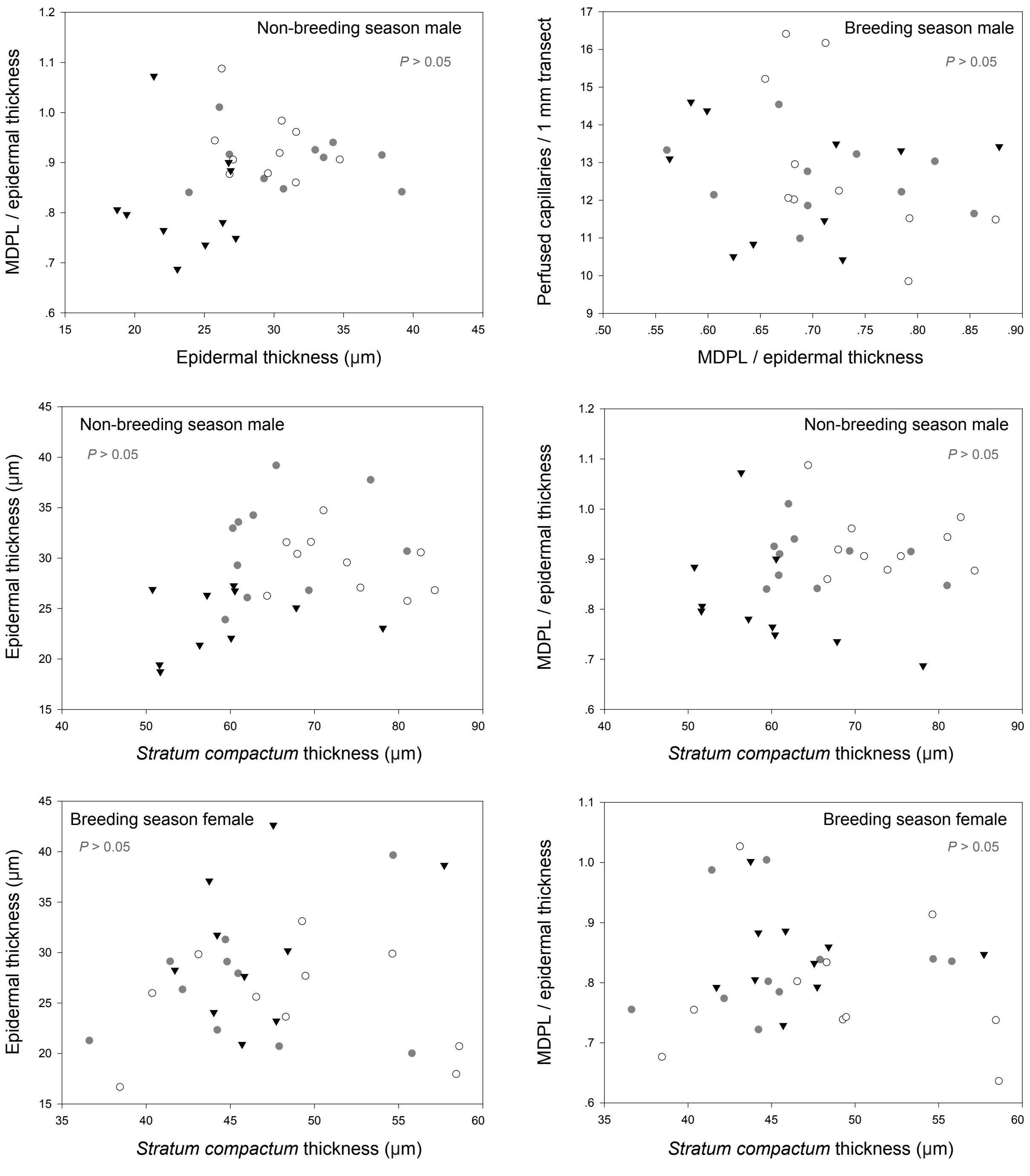

Figure S1 Plots between the extent of capillaries penetrating the epidermis, thickness of the epidermis, perfused subepidermal capillary density, and *stratum compactum* thickness across ten body regions in *Leptobrachium boringii*. Symbols are used to distinguish individuals of the same group, and regional mean values are plotted. The *P* values were derived from generalized linear mixed models where individual was included as a random factor. MDPL: average minimum diffusion path length of subepidermal capillaries.
